# Relationship between oral health literacy and oral frailty among older adults in nursing homes: a latent profile analysis

**DOI:** 10.3389/fpubh.2026.1804760

**Published:** 2026-05-28

**Authors:** Yang Zhao, Ting-Ting Wang, Ling-Na Kong, Jun Yang, Fen Xie, Ying Huang, Qiao-Ling Yu, Hua-Yan Ran

**Affiliations:** 1 Department of Nursing, The First Affiliated Hospital of Chongqing Medical University, Chongqing, China; 2 School of Nursing, Chongqing Medical University, Chongqing, China; 3 Department of General Practice, The First Affiliated Hospital of Chongqing Medical University, Chongqing, China

**Keywords:** latent profile analysis, nursing homes, older adults, oral frailty, oral health literacy

## Abstract

**Objectives:**

To identify different profiles of oral health literacy among older adults in nursing homes and to explore the relationship between these profiles and oral frailty.

**Design:**

A cross-sectional study.

**Methods:**

A convenience sample of 403 older adults living in nursing homes was recruited between September and December 2024. Participants completed a general information questionnaire, the Oral Frailty Index-8, and the Short-Form Health Literacy in Dentistry scale. Latent profile analysis was applied to identify oral health literacy profiles. Logistic regression was used to examine the relationship between oral health literacy profiles and oral frailty.

**Results:**

Three oral health literacy profiles were identified: ‘low oral health literacy-low understanding group’ (22.1%), ‘moderate oral health literacy-low communication group’ (46.3%), and ‘high oral health literacy-high economic group’ (31.6%). After adjustment for potential confounders, oral health literacy profiles were significantly associated with oral frailty. Participants in the ‘high oral health literacy-high economic group’ had a significantly lower risk of oral frailty compared with the other two groups.

**Conclusion:**

Oral health literacy among older adults in nursing homes showed significant heterogeneity, with three distinct latent profiles. These profiles were significantly associated with oral frailty. Tailored interventions based on oral health literacy profiles may help delay oral frailty among older adults in nursing homes.

## 1 Introduction

Oral frailty refers to a series of age-related deteriorations in oral health and is associated with an increased risk of adverse health outcomes, including physical frailty, falls, hospitalization, disability, and mortality (1, 2). Oral frailty can be manifested by multiple poor oral health indicators, encompassing tooth loss, poor oral hygiene, periodontal disease, xerostomia, oral pain, and difficulties in chewing or swallowing (2). These oral health problems are particularly pronounced among older adults living in nursing homes, partly due to their diminished self-care capacity and complex health needs (3). A study reported that 46.1% of older adults in nursing homes had no natural teeth, 44.0% had untreated caries with pulp involvement, and 68.5% had poor denture hygiene (4). However, oral health problems among older adults in nursing homes are often overlooked. This may be due to shortages of nursing staff, limited time for carers to provide oral care, and the low prioritization of oral care in daily routines (5). Consequently, older adults in nursing homes face an increased risk of oral frailty and its related adverse health outcomes. The prevalence of oral frailty among older adults in nursing homes has been reported to be 53.0% (1), highlighting the need for early, targeted prevention and intervention.

Oral health literacy (OHL) is important for preventing oral frailty. OHL refers to the ability to obtain, understand, and use essential oral health information and services, enabling individuals to make informed decisions about their oral health (6). Previous evidence has consistently shown that higher OHL levels are associated with favorable oral health behaviors, such as regular toothbrushing, flossing, routine dental visits, and actively seeking oral health information (7, 8). The negative association between oral health behaviors and oral frailty has been observed in older adults receiving maintenance hemodialysis (9). Besides, individuals with higher OHL tend to exhibit more favorable oral health outcomes, including better oral hygiene, greater retention of natural teeth, and less severe periodontitis (8, 10). These oral health outcomes are also key indicators of oral frailty (11). Moreover, a survey of older adults with ischemic stroke found that OHL was a predictor of oral frailty (12), demonstrating a direct link between OHL and oral frailty. Thus, enhancing OHL may be a promising approach to preventing oral frailty. However, evidence on the impact of OHL on oral frailty remains limited, particularly in nursing home settings. Clarifying the relationship between OHL and oral frailty among older adults in nursing homes may help inform targeted prevention strategies.

OHL comprises multiple dimensions, including communication, receptivity, understanding, support, economics, utilization, and accessibility (13). Individuals may perform differently across these dimensions. In addition, OHL is influenced by various factors, including age, education level, economic status, and access to healthcare resources (6, 14), suggesting its heterogeneity across populations. However, most existing studies have classified individuals into different OHL levels based solely on the total scores of measurement tools (10, 15), which may overlook both the multidimensional nature of OHL and population heterogeneity. Latent profile analysis (LPA), a person-centered statistical method, provides a suitable alternative by classifying individuals into homogeneous subgroups based on continuous variables, such as dimensional scores from scales (16). Applying LPA may help identify distinctive OHL profiles and their sociodemographic characteristics, thereby supporting more precise and tailored interventions. Therefore, this study aimed to use LPA to identify latent profiles of OHL among older adults in nursing homes and to examine their relationship with oral frailty, thereby informing targeted strategies to enhance OHL and reduce the risk of oral frailty.

## 2 Methods

### 2.1 Study design and participants

A cross-sectional study was conducted. Participants were recruited from nine nursing homes in Chongqing, China, using convenience sampling. The inclusion criteria were: (1) age ≥ 60 years, (2) length of stay ≥ 90 days, (3) clear consciousness and the ability to communicate verbally, and (4) informed consent. Exclusion criteria included a diagnosis of dementia, communication difficulties, or other severe health conditions that could interfere with the completion of the questionnaires. Prior research suggests that latent profile analysis can yield stable solutions with sample sizes of approximately 300 or above (17). Our sample size of 403 was adequate for analysis.

### 2.2 Instruments

#### 2.2.1 General information questionnaire

The research team developed a general information questionnaire based on a review of previous literature. The questionnaire included gender (male, female), age (60–79, 80–89, ≥ 90 years), education level (primary school or below, middle or high school, and college or university), individual income (< 3,000, 3,000-5,000, > 5,000 yuan/month), residential size (≤ 100, 101–399, ≥ 400 persons), smoking status (never, former or current), number of remaining teeth (< 20, ≥ 20 teeth) and toothbrushing frequency (< 2, ≥ 2 times/day). The categorization of variables was based on commonly used thresholds in geriatric and oral health research.

#### 2.2.2 Short-Form Health Literacy in Dentistry scale (HeLD-14)

OHL was assessed using the HeLD-14 developed by Jones et al. (13). It consists of 14 items covering seven dimensions: communication, understanding, receptivity, utilization, support, economics, and access. Each item is rated on a 5-point Likert scale, ranging from 0 (unable to do) to 4 (without any difficulty). The total score ranges from 0 to 56, with higher values indicating better OHL. The HeLD-14 has demonstrated satisfactory reliability (Cronbach’s *α* = 0.908) and structural validity (18). In the present study, Cronbach’s α was 0.952.

#### 2.2.3 Oral Frailty Index-8 (OFI-8)

Oral frailty was measured using the OFI-8 developed by Tanaka et al. (19). It comprises eight items assessing five dimensions: denture use, swallowing function, social participation, oral health-related behaviors, and chewing ability. The total score ranges from 0 to 11, with scores ≥ 4 indicating oral frailty and higher scores reflecting worse oral frailty. The Cronbach’s *α* for OFI-8 was 0.692 in the original study (19), and it was 0.608 in this study. The difference in Cronbach’s α may be partly explained by the multidimensional nature of the OFI-8 and the heterogeneity of older adults in nursing homes. Specifically, older adults in nursing homes may vary considerably in oral function, oral health-related behaviors, and social participation, which may have reduced inter-item correlations, thereby contributing to the lower Cronbach’s *α* observed in this study compared with the original study.

### 2.3 Data collection

Data were collected between September and December 2024. Three trained investigators conducted all data collection through one-to-one interviewer-administered surveys with the assistance of nursing home staff. Nursing home staff only helped identify eligible participants and coordinate survey arrangements. They did not interpret questionnaire items, explain participants’ answers, or record responses. Before data collection, the investigators received standardized training from the project leader to ensure consistent administration of all assessment tools. Investigators screened and recruited eligible older adults according to the inclusion criteria. Participants who agreed to join the study provided written informed consent. During the survey, investigators read each questionnaire item aloud and recorded the participants’ answers directly on the form. Standardized instructions were used to ensure consistency across interviews. To protect privacy and reduce potential reporting bias, interviews were conducted in a private room whenever possible, with only the investigator and the participant present. Participants were informed that their responses would remain confidential and inaccessible to nursing home staff. All completed questionnaires were checked on-site immediately after the survey to ensure accuracy and completeness.

### 2.4 Ethical considerations

The study protocol adhered to the principles of the Declaration of Helsinki and was approved by the institutional ethics committee (Approval number: 2024–152-01). All participants were informed of the study’s objectives and the anonymity and confidentiality of their responses before enrolling. All participants gave informed consent before data collection and could withdraw at any time.

### 2.5 Data analysis

Data analyses were performed using SPSS 25.0 and Mplus 8.3. LPA was conducted based on the scores of the seven dimensions of the HeLD-14. Models with one to five latent profiles were fitted. To reduce the risk of local maximum solutions, random starts were specified in Mplus using STARTS = 200 50, indicating 200 initial-stage random starts and 50 final-stage optimizations. The evaluation indices of model fit included: (1) Akaike Information Criteria (AIC), Bayesian Information Criteria (BIC), and adjusted Bayesian Information Criteria (aBIC), with smaller values indicating better model fit; (2) entropy, ranging from 0 to 1, with values closer to 1 indicating better model fit and values above 0.8 reflecting classification accuracy over 90%; and (3) the Bootstrap Likelihood Ratio Test (BLRT) and the Lo–Mendell–Rubin (LMR), with *p*-values < 0.05 indicating that the model with k profiles fits significantly better than the model with k-1 profiles (17, 20). The optimal latent profile model was determined based on a comprehensive evaluation of these statistical criteria and the interpretability of the identified profiles (17).

Categorical variables were presented as frequencies and percentages, and group differences were assessed using the chi-square test. Continuous variables with approximately normal distributions were expressed as mean and standard deviation (SD); analysis of variance (ANOVA) was used when variances were homogeneous (Bonferroni *post hoc* tests), and Welch’s ANOVA was applied when variances were unequal (Games-Howell *post hoc* tests). Continuous variables with skewed distributions were expressed as median and interquartile range (IQR) and compared between groups using the Kruskal-Wallis H test, followed by Bonferroni correction for *post hoc* testing. Based on previous literature, gender, age, education level, individual income, smoking status, number of remaining teeth, and toothbrushing frequency were selected as candidate covariates (1, 6, 9, 21). In addition, residential size was included as a candidate covariate because it was considered a contextual characteristic related to care organization and resource allocation across nursing homes in the Chinese context (22). Univariate logistic regression was first performed to examine the associations between these candidate covariates and oral frailty (no = 0, yes = 1). Variables with *p* < 0.10 in the univariate analyses were then included as covariates in the multivariable logistic regression model to examine the association between OHL profiles and oral frailty. Multicollinearity among the selected variables was assessed using tolerance and variance inflation factors (VIF). Crude odds ratios (cOR), adjusted odds ratios (aOR), and 95% confidence intervals (CI) were reported. A *p*-value < 0.05 (two-tailed) was considered statistically significant.

## 3 Results

### 3.1 Participants’ characteristics

Among the 410 eligible participants, 403 (98.2%) consented to participate and completed the questionnaire. The mean age of the participants was 84.64 years (SD 6.67), with a range of 62 to 99 years. Most participants were aged 80–89 years (53.8%), female (62.0%), had a primary school education or below (47.1%), and had an individual monthly income of 3,000–5,000 yuan (60.3%). A total of 34.0% of participants resided in nursing homes with 101–399 persons. About 59.6% of participants had fewer than 20 remaining teeth, 38.2% brushed their teeth < 2 times/day, and 17.6% were former or current smokers. The prevalence of oral frailty was 68.2%. The mean scores of oral frailty and OHL were 5.13 (SD 2.66) and 31.51 (SD 11.84), respectively.

### 3.2 Latent profiles of OHL

A total of five latent profile models were fitted in this study. The fit indices for each model are presented in Table 1. The AIC, BIC, and aBIC values gradually decreased with the number of profiles, indicating improved model fit. The *p*-value of the LMR for Models 4 and 5 was not statistically significant (*p* > 0.05), leading to their exclusion. Model 3 showed the highest entropy (0.919), indicating strong classification accuracy. Thus, Model 3 was selected as the optimal model. The average posterior probabilities of the three profiles in Model 3 were 94.7, 95.3, and 95.8%, respectively, suggesting that the classification was reliable when three latent profiles were specified.

**Table 1:** Fitting indices of different latent profile models.

Model	AIC	BIC	aBIC	Entropy	BLRT	LMR	Profile proportion (%)
					*p* value	*p* value	
1	11886.707	11942.692	11898.269				
2	10682.846	10770.823	10701.015	0.897	< 0.001	0.007	31.6/68.4
**3**	**10066.275**	**10186.243**	**10091.050**	**0.919**	**< 0.001**	**< 0.001**	**22.1/46.3/31.6**
4	9835.495	9987.454	9866.877	0.915	< 0.001	0.234	18.2/43.7/8.0/30.1
5	9744.634	9928.585	9782.622	0.879	< 0.001	0.111	18.1/10.0/28.2/21.0/22.7

aBIC, adjusted BIC; AIC, Akaike information criterion; BIC, Bayesian information criterion; BLRT, Bootstrap likelihood ratio test; LMR, Lo–Mendell–Rubin. The bold values are the preferred model.

### 3.3 Naming of three latent profiles of OHL

**Figure 1:**
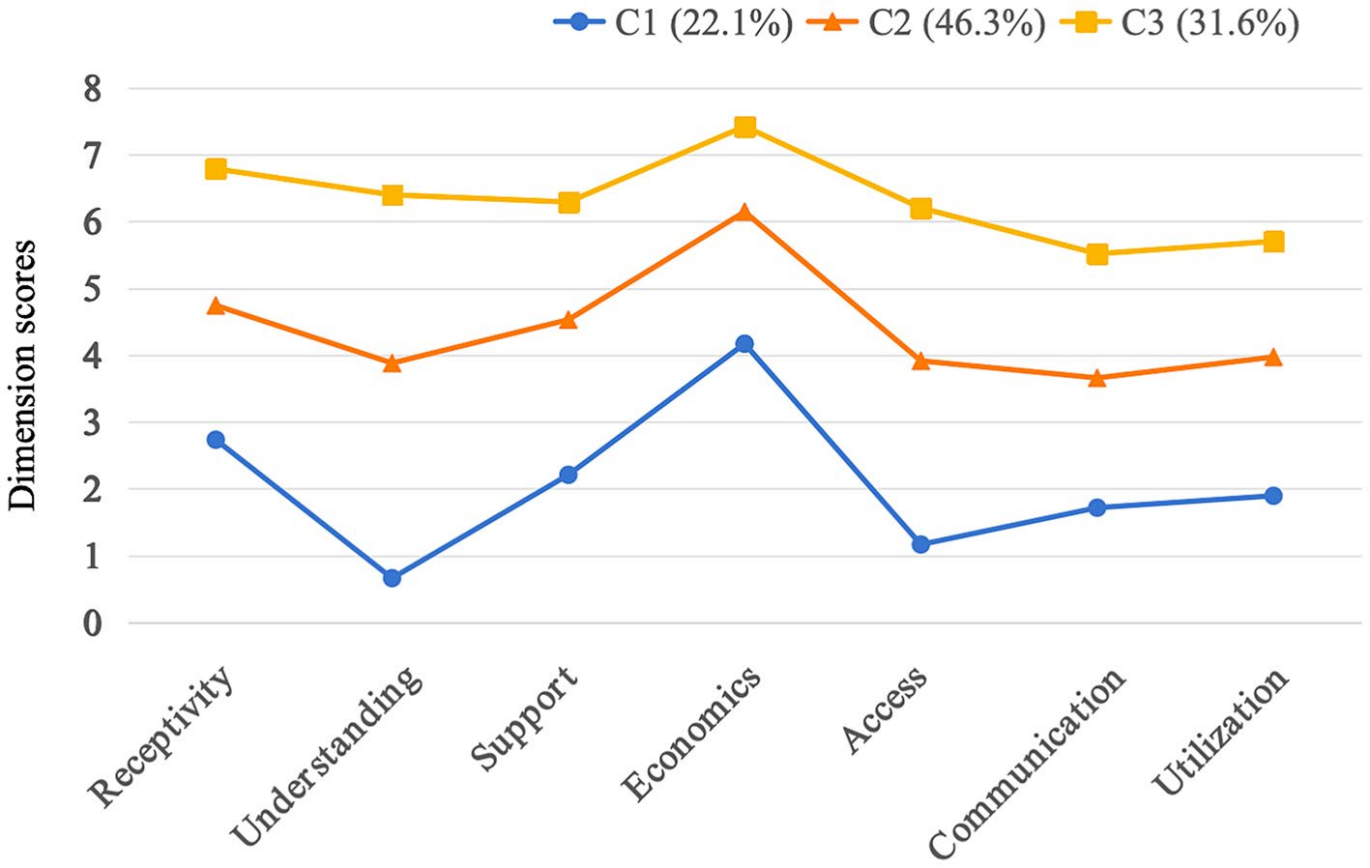
Three latent profiles of oral health literacy among older adults in nursing homes. C1, low OHL-low understanding group; C2, moderate OHL-low communication group; C3, high OHL-high economic group.

Figure 1 shows the mean scores of the seven dimensions of the HeLD-14 across the three OHL latent profiles among older adults in nursing homes. The C1 group (*n* = 89, 22.1%) had the lowest overall OHL score (mean 14.49, SD 6.68), with consistently lower scores across all dimensions than those of C2 and C3, particularly in understanding. Therefore, this group was labeled ‘low OHL-low understanding group’. The C2 group (*n* = 187, 46.3%) showed a moderate OHL score (mean 30.89, SD 4.16), with relatively low scores in communication, and was labeled ‘moderate OHL-low communication group’. The C3 group (*n* = 127, 31.6%) exhibited the highest OHL score (mean 44.35, SD 4.44), with higher scores than those of C1 and C2 across all dimensions, especially in economics. Thus, this group was labeled ‘high OHL-high economic group’.

### 3.4 Comparison of participants’ characteristics among the three latent profiles of OHL

Table 2 displays the characteristics of the participants based on the three latent profiles of OHL. Chi-squared tests revealed significant differences among the three groups in gender, education level, individual income, residential size, number of remaining teeth, and toothbrushing frequency (*p* < 0.05), whereas no significant differences were found in age and smoking status (*p* > 0.05). Compared with the C1 and C2 groups, the C3 group had a higher proportion of females (68.5%), middle school or above (86.6%), monthly income > 5,000 yuan (30.7%), residential size ≥ 400 persons, remaining teeth ≥ 20 teeth (68.5%), and toothbrushing frequency ≥ 2 times/day (87.4%). In contrast to the C2 and C3 groups, the C1 group had a higher percentage of males (48.3%), primary school or below (94.4%), monthly income < 3,000 yuan (65.2%), residential size ≤ 100 persons (70.8%), remaining teeth < 20 teeth (79.8%) and toothbrushing frequency < 2 times/day (75.3%). The C2 group had a higher percentage of monthly income of 3,000–5,000 yuan (71.7%) and residential size of 101–399 persons (43.9%) than the C1 and C3 groups.

**Table 2:** Comparison of participants’ characteristics in three latent profiles of OHL.

Variables	*N* (%)	C1 (*n* = 89)	C2 (*n* = 187)	C3 (*n* = 127)	*χ*^2^	*p* value
Age (years)					6.737	0.150
60–79	81 (20.1)	24 (27.0)	37 (19.8)	20 (15.7)		
80–89	217 (53.8)	40 (44.9)	99 (52.9)	78 (61.5)		
≥ 90	105 (26.1)	25 (28.1)	51 (27.3)	29 (22.8)		
Gender					6.327	**0.042**
Male	153 (38.0)	43 (48.3)	70 (37.4)	40 (31.5)		
Female	250 (62.0)	46 (51.7)	117 (62.6)	87 (68.5)		
Education					140.953	**< 0.001**
≤ Primary school	190 (47.1)	84 (94.4)	89 (47.6)	17 (13.4)		
Middle/high school	170 (42.2)	5 (5.6)	82 (43.8)	83 (65.4)		
College and university	43 (10.7)	0 (0.0)	16 (8.6)	27 (21.2)		
Individual income (yuan/month)					133.879	**< 0.001**
< 3,000	112 (27.8)	58 (65.2)	44 (23.5)	10 (7.9)		
3,000–5,000	243 (60.3)	31 (34.8)	134 (71.7)	78 (61.4)		
> 5,000	48 (11.9)	0 (0.0)	9 (4.8)	39 (30.7)		
Residential size (person)					111.852	**< 0.001**
≤ 100	130 (32.3)	63 (70.8)	49 (26.2)	18 (14.2)		
101–399	137 (34.0)	21 (23.6)	82 (43.9)	34 (26.8)		
≥ 400	136 (33.7)	5 (5.6)	56 (29.9)	75 (59.0)		
Smoking					1.611	0.447
Never	332 (82.4)	71 (79.8)	152 (81.3)	109 (85.8)		
Former/current	71 (17.6)	18 (20.2)	35 (18.7)	18 (14.2)		
Number of remaining teeth (teeth)					63.519	**< 0.001**
< 20	240 (59.6)	71 (79.8)	129 (69.0)	40 (31.5)		
≥ 20	163 (40.4)	18 (20.2)	58 (31.0)	87 (68.5)		
Toothbrushing frequency (times/day)					87.090	**< 0.001**
≥ 2	249 (61.8)	22 (24.7)	116 (62.0)	111 (87.4)		
< 2	154 (38.2)	67 (75.3)	71 (38.0)	16 (12.6)		
Oral frailty					78.114	**< 0.001**
No	128 (31.8)	10 (11.2)	40 (21.4)	78 (61.4)		
Yes	275 (68.2)	79 (88.8)	147 (78.6)	49 (38.6)		

OHL, oral health literacy; C1, low OHL-low understanding group; C2, moderate OHL-low communication group; C3, high OHL-high economic group. The bold values are *p* < 0.05.

### 3.5 Relationship between the three OHL profiles and oral frailty

#### 3.5.1 Differences in oral frailty within the three OHL profiles

There were statistically significant differences in the prevalence of oral frailty among the three OHL profiles (*χ*^2^ = 78.114, *p* < 0.05; see Table 2). The prevalence was highest in the C1 group (88.8%), followed by the C2 group (78.6%) and lowest in the C3 group (38.6%). Significant differences were also observed in OFI-8 total score and dimension scores across the three groups (all *p* < 0.05; see Table 3). Overall, the C1 group had the highest OFI-8 scores (mean 6.13, SD 2.16), whereas the C3 group had the lowest scores (mean 3.36, SD 2.10). In pairwise comparisons, participants in the C3 group had significantly lower scores for the OFI-8 total scores and its dimension scores (chewing, swallowing, social participation, and oral health-related behaviors) than those in the C1 and C2 groups (*p* < 0.05). No significant differences were found between the C1 and C2 groups in the OFI-8 total and dimension scores for chewing, swallowing, and social participation (*p* > 0.05), nor between the C1 and C3 groups in denture scores (*p* > 0.05).

**Table 3:** Comparison of oral frailty scores of participants in three latent profiles of OHL.

Variables	Total score	C1	C2	C3	*F*/*H*	*p* value			
						Overall	C3 vs. C2	C3 vs. C1	C2 vs. C1
OFI-8 score	5.13 (2.66)	6.13 (2.16)	5.85 (2.65)	3.36 (2.10)	60.499^a^	**< 0.001**	**< 0.001**	**< 0.001**	0.610
Chewing	1.76 (1.34)	2.27 (1.15)	2.06 (1.23)	0.96 (1.28)	39.198^a^	**< 0.001**	**< 0.001**	**< 0.001**	0.364
Swallowing	0.00 (0.00, 1.00)	1.00 (0.00, 1.00)	0.00 (0.00, 1.00)	0.00 (0.00, 0.00)	32.836^b^	**< 0.001**	**< 0.001**	**< 0.001**	1.000
Denture	0.00 (0.00, 2.00)	0.00 (0.00, 2.00)	2.00 (0.00, 2.00)	0.00 (0.00, 2.00)	18.240^b^	**< 0.001**	**0.006**	0.864	**< 0.001**
Social participation	0.43 (0.50)	0.54 (0.50)	0.46 (0.50)	0.30 (0.46)	7.478^a^	**0.001**	**0.010**	**0.001**	0.436
Oral health-related behavior	1.31 (0.57)	1.75 (0.43)	1.32 (0.53)	0.97 (0.49)	77.075^a^	**< 0.001**	**< 0.001**	**< 0.001**	**< 0.001**

C1, low OHL-low understanding group; C2, moderate OHL-low communication group; C3, high OHL-high economic group; OFI-8, oral frailty index-8; OHL, oral health literacy. *Post hoc* test: Games-Howell test for OFI-8 score, chewing, social participation and oral health-related behavior; Bonferroni correction for swallowing and denture. The bold values are *p* < 0.05.

a Welch’s ANOVA test.

b Kruskal-Wallis test.

#### 3.5.2 Logistic regression analysis of oral frailty

Table 4 shows the results of both univariate and multivariate logistic regression analyses. In univariate regression, age, gender, education level, individual income, residential size, smoking status, number of remaining teeth, toothbrushing frequency, and OHL latent profiles were associated with oral frailty (*p* < 0.10). All these variables had tolerance values > 0.10 and VIF values < 5, indicating no multicollinearity. In the multivariable regression model, after adjusting for variables identified in the univariate analyses (*p* < 0.10), participants in the C1 group (aOR = 13.14, 95% CI: 3.00–57.52) and the C2 group (aOR = 4.97, 95% CI: 1.93–12.80) had significantly higher odds of oral frailty compared with the C3 group.

**Table 4:** Factors associated with oral frailty.

Variables	Oral frailty	*p* value	Oral frailty	*p* value
	cOR (95%CI)		aOR (95%CI)	
Age (years)		0.012*		**0.007**
80–89	1.36 (0.80, 2.29)		1.95 (0.75, 5.07)	
≥ 90	2.59 (1.36, 4.95)		6.19 (1.94, 19.77)	
Gender		0.020*		0.190
Female	0.58 (0.38, 0.92)		0.51 (0.19, 1.39)	
Education		0.001*		**0.001**
≤ Primary school	2.46 (1.23, 4.93)		0.31 (0.07, 1.36)	
Middle/high school	1.11 (0.56, 2.18)		0.06 (0.01, 0.34)	
Individual income (yuan/month)		< 0.001*		**< 0.001**
< 3,000	50.60 (16.31, 156.96)		16.86 (3.11, 91.49)	
3,000–5,000	30.77 (10.63, 89.03)		19.27 (4.87, 76.34)	
Residential size (person)		0.051*		0.375
≤ 100	1.92 (1.12, 3.26)		0.51 (0.17, 1.54)	
101–399	1.19 (0.72, 1.95)		0.53 (0.20, 1.43)	
Smoking		0.036*		0.147
Former/current	1.92 (1.04, 3.56)		0.38 (0.11, 1.39)	
Number of remaining teeth (teeth)		< 0.001*		**< 0.001**
< 20	60.31 (29.35, 123.94)		55.79 (22.56, 137.93)	
Toothbrushing frequency (times/day)		< 0.001*		**0.002**
< 2	5.13 (3.01, 8.74)		4.19 (1.71, 10.32)	
Latent profiles of OHL		< 0.001*		**0.001**
Low OHL-low understanding group	12.58 (5.95, 26.59)		13.14 (3.00, 57.52)	
Moderate OHL-low communication group	5.85 (3.55, 9.64)		4.97 (1.93, 12.80)	

aOR, adjusted odds ratio; CI, confidence intervals; cOR, crude odds ratio; OHL, oral health literacy. *Included in multivariate logistic regression analysis (*p* < 0.10). The bold values are *p* < 0.05.

## 4 Discussion

To our knowledge, this was the first study to apply LPA to identify heterogeneous subtypes of OHL among older adults in nursing homes. Three distinct latent profiles were identified, namely the ‘low OHL-low understanding group’, the ‘moderate OHL-low communication group’, and the ‘high OHL-high economic group’. Significant differences were observed in demographic and OHL characteristics among the three groups. Moreover, OHL profiles were significantly associated with oral frailty in this population, with participants in the ‘high OHL-high economic group’ having significantly lower risks of oral frailty compared with those in the other two groups. These findings may help healthcare providers in nursing homes develop targeted interventions to improve OHL among older adults and reduce their risk of oral frailty.

The ‘low OHL-low understanding group’ accounted for 22.1% of participants and showed the lowest OHL level among the three profiles, particularly in the understanding dimension. This pattern is likely related to their disadvantaged socioeconomic background, including low education, limited income, and less favorable living conditions, which are established correlates of inadequate OHL (15, 23). Specifically, low educational attainment may impair the ability to comprehend oral health information. Economic hardship can restrict access to dental services and preventive resources (21). In addition, many small-scale nursing homes lack regular support from dental professionals, standardized oral care procedures, and adequate oral hygiene supplies (5), further reducing opportunities for receiving professional oral health guidance. Consistent with this interpretation, individuals in this group demonstrated poorer oral health behaviors and outcomes. For this group, it is crucial to provide clear, easy-to-understand educational materials, regular support from dental professionals, and improved access to oral hygiene resources to strengthen basic understanding and to encourage preventive behaviors.

The ‘moderate OHL-low communication group’ accounted for nearly half of our sample (46.3%), with a moderate OHL level. This suggests that they have the basic ability to receive, understand, and process oral health information and may benefit substantially from targeted interventions. However, older adults in this group performed relatively poorly in the communication dimension, which may stem from contextual and interactional barriers. In nursing homes, caregivers are the primary providers of daily care for older adults. However, many of them lack adequate training and skills in oral health care (5), which may reduce opportunities for oral health-related communication during daily care. In addition, older adults in nursing homes may have limited dental visits due to physical or transportation barriers (24), which may limit their familiarity with dental care environments and terminology, thereby hindering effective communication with dentists (25). Tailored interventions for this group should focus on improving oral health-related communication in daily care. Based on patient-centered dental communication strategies, nursing homes could provide communication-skills training for caregivers, including encouraging questions and feedback, using visual aids, and applying active listening and summaries to enhance communication with older adults (25). Older adults should also be encouraged to express oral discomfort and unmet dental care needs during daily care, thereby supporting two-way communication with caregivers.

The ‘high OHL-high economic group’ (31.6% of participants) exhibited the highest overall OHL, along with more favorable economic conditions, oral health behaviors and outcomes. These findings may suggest a reinforcing pattern whereby greater socioeconomic resources are associated with higher OHL (6), which in turn supports more consistent engagement in preventive oral health behaviors and contributes to more favorable oral health outcomes (26). For this group, selected digital health technologies may be used to support sustained improvements in OHL, such as mobile applications, web-based platforms, or webinars (27). Furthermore, individuals in this group could serve as a valuable peer resource to support older adults with lower OHL by sharing effective oral health behaviors and encouraging engagement in preventive practices.

Based on between-group comparisons across oral frailty dimensions among the three OHL profiles, the ‘high OHL-high economic group’ demonstrated more favorable performance in core oral functions, such as chewing and swallowing, and showed higher levels of social participation. One possible explanation is that higher OHL may encourage greater awareness of oral health and more proactive preventive dental care use, which together could contribute to better maintenance of oral function (26, 28). In contrast, the observed difference in social participation should be interpreted with caution, because we did not examine the independent association between OHL and social participation, and direct evidence directly linking these two constructs remains limited.

Our findings indicate that OHL profiles were independently associated with oral frailty among older adults in nursing homes. This is consistent with previous research in older adults with ischemic stroke, which identified OHL as an independent predictor of oral frailty (12). Several pathways may explain this association. First, individuals with inadequate OHL often have limited awareness of oral diseases and may fail to recognize early signs of periodontal inflammation, caries, or functional decline (21), allowing minor oral problems to accumulate over time and ultimately contribute to the development of oral frailty (11). Second, limited OHL may restrict individuals’ utilization of dental services, which could contribute to delayed or missed care and further exacerbate oral health problems, thereby increasing the risk of oral frailty (8, 29). In addition, lower OHL may be associated with reduced confidence and self-efficacy in maintaining daily oral hygiene, potentially limiting effective self-care behaviors and increasing vulnerability to oral frailty (26, 30). Notably, given the cross-sectional design of this study, the possibility of a bidirectional relationship should also be considered. Previous evidence has shown that oral frailty is associated with declines in physical and psychological function, social withdrawal, and reduced social participation among older adults (31). These problems may weaken older adults’ interest and motivation in oral health and limit their opportunities to obtain, communicate, and use oral health information, thereby potentially affecting their OHL (32). Together, these findings highlight the importance of strengthening OHL and early identification of oral frailty as part of routine oral health management for older adults in nursing homes. Targeted oral health education, improved access to dental services, and reinforcement of oral health-related self-efficacy may help prevent the progression of minor oral problems and interrupt the potential cycle between inadequate OHL and oral frailty.

There were some limitations in this study. First, the cross-sectional survey limited causal inference between the OHL and oral frailty. Longitudinal studies are needed to confirm the temporal relationship. Second, this study used convenience sampling and included only older adults from nine specific nursing homes in Chongqing, China, which may restrict the generalizability of the findings to older adults from other geographical regions and residential settings. Future multicenter studies using more rigorous sampling strategies and larger, more diverse samples are warranted. Last, the OFI-8 showed lower internal consistency than in the original study, and both HeLD-14 and OFI-8 were self-reported measures. Future studies should consider using more robustly validated oral frailty tools for nursing home populations and combining self-reported measures with objective clinical assessments.

## 5 Conclusion

Our study showed that there was significant heterogeneity in OHL among older adults in nursing homes, which can be classified as ‘low OHL-low understanding group’, ‘moderate OHL-low communication group’, and ‘high OHL-high economic group’. Different OHL latent profiles were significantly associated with oral frailty among older adults in nursing homes. Identifying OHL subgroups provides a practical basis for targeted oral health management in nursing homes. Routine assessment of OHL and the development of tailored interventions for different profiles may enhance OHL and reduce the risk of oral frailty among older adults in nursing homes. Future studies should further examine the effectiveness of OHL profile-based interventions in reducing oral frailty and its adverse health outcomes.

## Data Availability

The original contributions presented in the study are included in the article/supplementary material, further inquiries can be directed to the corresponding author.
